# When is the best time to test paratuberculosis positivity? Observations from a follow-up study in Hungarian dairy herds

**DOI:** 10.3389/fvets.2025.1570915

**Published:** 2025-06-19

**Authors:** Barbara Vass-Bognár, Mikolt Bakony, Kinga Fornyos, Walter Baumgartner, Johannes Lorenz Khol, Viktor Jurkovich

**Affiliations:** ^1^Department of Animal Hygiene, Herd Health and Mobile Clinic, University of Veterinary Medicine, Budapest, Hungary; ^2^Centre for Translational Medicine, Semmelweis University, Budapest, Hungary; ^3^Eurofins Vet-Controll Ltd., Budapest, Hungary; ^4^Clinical Center for Ruminant and Camelid Medicine, Clinical Department for Farm Animals and Food System Science, University of Veterinary Medicine, Vienna, Austria; ^5^Centre for Animal Welfare, University of Veterinary Medicine, Budapest, Hungary

**Keywords:** dairy cow, paratuberculosis, ELISA, qPCR, sampling time

## Abstract

The objective of the present study was to find the most practical combination of diagnostic procedures and time points during lactation to identify *Mycobacterium avium* ssp. *paratuberculosis* (MAP)-infected animals. Four Hungarian dairy farms with a 4–5% apparent MAP positivity were enrolled in the study, and 13 non-lactating, known MAP-positive pregnant cows were chosen from each farm. Feces, blood, and milk samples were collected from each cow at 1–5, 10–14, 40–60, 90–120, 180–200, and 280–300 days in milk (DIM) and ELISA and PCR assays were performed for antibody or pathogen detection. Animals that later developed clinical paratuberculosis symptoms showed distinctly different patterns of test positivity than those that did not develop clinical symptoms during the observation period. The optimal time for detecting MAP-positive animals with the highest probability was DIM 40–60 with serum ELISA and DIM 10–14 and 40–60 for PCR assays, respectively. Serum ELISA proved to be slightly more sensitive than milk ELISA. S/P values showed a moderate correlation with the fecal qPCR Ct values. We found that the most suitable period for MAP screening is 40–60 days after calving.

## 1 Introduction

Bovine paratuberculosis (Johne’s disease; JD) is a chronic intestinal disease caused by *Mycobacterium avium* ssp. *paratuberculosis* (MAP). Paratuberculosis adversely affects dairy cows’ health, milk yield, and slaughter value (1), so JD is a significant production-limiting disease worldwide (2). For this reason, control programs are considered highly necessary for dairy farming and food hygiene management (3–6).

Fecal-oral contamination is the most important way of paratuberculosis transmission, with the incubation period extending from 2 to 10 years, influenced by the infecting dose (7). From a clinical point of view, the disease can be classified into four stages, in which the degree of bacterial shedding and the dynamics of the immune response vary (8–10). There is no effective treatment for paratuberculosis (11), and a link between MAP in Crohn’s disease in humans is suspected but has not yet been identified (12–14); therefore, eradication programs were established in many countries (2, 15, 16). These programs are based on two main strategies to control the spread of MAP in a herd. The first essential element of paratuberculosis eradication programs is the immediate removal of the calf from its mother and the feeding of MAP-free colostrum and milk, limiting MAP transmission to calves (17). The second central element is the removal of MAP-infected cows from the herd (18, 19) by a so-called test-and-cull procedure. Culturing MAP from feces has been considered the gold standard for diagnosing paratuberculosis, however MAP culture incubation can take up to 4 weeks on broth media (20) and 16 weeks on solid media (21). The positive predictive value of MAP culture is almost 100%, however, sensitivity is 70% for animals showing clinical signs, but only 23–29% for subclinical infection. More rapid and cost-efficient PCR-based molecular techniques can detect MAP genetically, and studies have found that direct PCR has a similar sensitivity and specificity to culture (22). Therefore, the PCR replaced bacterial culture in most eradication and control programs. The cycle threshold (Ct) values obtained can be used to estimate the quantity of MAP DNA in the sample, with lower Ct values indicating higher MAP DNA concentration (23). Antibody detection methods, like serum or milk ELISA, are less costly and more rapid, however, serological methods are usually lower in sensitivity but high in specificity (24, 25). Interpreting ELISA S/P values may be informative of MAP infection severity, as S/P values greatly exceeding the detection thresholds would indicate a pronounced immune response and consequently a higher level of antigen stimulus (26). The milk ELISA is nearly similar to serum ELISA in terms of testing time and costs (27). The reliability of infection detection improves when a combination of tests is used or when cows and herds are tested repeatedly (28). Most control and eradication programs are based on repeated ELISA testing of blood or milk, or testing feces by PCR or bacterial culture (5, 29, 30). Two consecutive positive samples within 2–6 months are a basis for a culling decision (31, 32). The exact culling time may be influenced by test value (Ct or S/P), presence or absence of clinical symptoms and actual milk yield (33). However, the success of the control programs often varies (34).

Several longitudinal studies have been conducted to elucidate associations between different diagnostic testing strategies. These studies have focused on the relationship between serum ELISA and fecal culture [e.g., (35–39)]. Although the relationship between diagnostic tests has been explored in previous studies, much of this research has been cross-sectional and was focused on dichotomous comparisons and levels of concordance between binary outcomes [e.g., (40, 41)]. More recent studies focused on the kinetics of antibodies (42) or fecal shedding patterns, respectively (43).

Due to multiple invisible losses associated with JD and because the disease is not self-limiting, farms should establish a control program that fits the daily routine, is cost-effective, and can keep the disease in control. MAP-positive animals are usually missed by single-time screening, indicating that the evaluation of MAP antibody status is needed repeatedly through the animal’s lifetime (42, 44).

With the follow-up of animals previously diagnosed twice as MAP seropositive and therefore would later become culled, we aimed to screen the potential changes in the seropositivity rate and fecal and milk PCR positivity throughout the lactation period. Our main objectives were to (1) identify the period of lactation where MAP-infected animals can be identified with the highest probability and (2) to identify the method with reasonably high sensitivity. (3) A third objective was to compare the agreement of different testing methods and to quantify any associations between the S/P values of the ELISA assays and copy numbers as expressed by Ct values of the PCR.

## 2 Materials and methods

### 2.1 Animals and samplings

Based on the results of the serum ELISA (Idexx Pourquier MAP antibody ELISA test kit; Idexx Laboratories Inc., Westbrook, ME, USA), large-scale dairy farms that were MAP-positive were enrolled in the study.

The ELISA tests were performed in the laboratory of Eurofins Vet-Controll Ltd. (Budapest, Hungary) as part of the Hungarian voluntary paratuberculosis control program. All results were used with the consent of the farms. Four farms [average herd size of 513 ± 237 (mean ± SD) cows, min. 284, max. 840] with a 4–5% apparent MAP positivity were selected for the study. The selection criteria were that the farms should be on the same level with the apparent seroprevalence (within 1% difference) and close to the laboratory to be able to sample and ship the samples to the laboratory on the same day. These farms followed a test and cull procedure of excluding double-positive cows from breeding by not inseminating them and culling at the end of lactation. Upon manifestation of clinical signs, animals are culled immediately. We chose 52 non-lactating, pregnant dairy cows (age: 4.1 + 0.8 yr.; lactation: 2.9 + 0.7) for the study (13 animals from each farm) 3 weeks before their expected calving. The inclusion criteria of the cows were: (1) multiparous animals (2nd or more calving at the time of selection); (2) two consecutive serum ELISA results taken in a 2–6 month interval in their previous lactation confirmed to be MAP-positive; (3) no clinical signs of JD; (4) no other severe health problems in the last lactation.

We collected individual feces, blood, and milk samples from the focal cows six times after calving between the following periods: 1–5, 10–14, 40–60, 90–120, 180–200, and 280–300 days postpartum (days in milk; DIM).

Fecal samples were taken from the rectum of the focal cows using sterile disposable gloves. We put the samples (around 50 g from each cow) in 250 mL sterile plastic tubes with a screw cap. At the same time, blood samples were taken into native serum syringes (Monovette, Sarstedt AG & Co. KG., Nümbrecht, Germany) from the tail vessels. Additionally, we took 10 mL of mixed milk from all four udder quarters from each animal into sterile plastic tubes after cleaning and disinfecting the teats with 70% alcohol. The feces, serum, and milk samples were immediately cooled at 4°C and transported to the laboratory within 2 h after sampling, where they were stored at −20°C until processing.

Body condition scoring [BCS; 1–5 scale; (45)] and fecal scoring were performed when the samples were collected. Fecal consistency was scored on a 1–5 scale with whole point precision, with 1 marking very thin, waterlike consistency and 5 marking very thick consistency (46).

During the study, 16 experimental animals were culled due to clinical manifestation of paratuberculosis between DIM 60 and 90. Three further animals were culled due to lameness issues and low production performance between 120 and 180 days DIM.

### 2.2 Laboratory procedures

The individual serum and milk samples were processed using the Idexx Pourquier MAP antibody ELISA test kit (Idexx Laboratories Inc., Westbrook, ME, USA) according to the manufacturer’s instructions. Optical density values were transformed to a sample-to-positive (S/P) ratio (26). Serum samples with an S/P ≥ 55% and milk samples with an S/P ≥ 30% were considered positive according to the manufacturer’s instructions, respectively.

DNA extraction from the fecal samples was performed using the Nucleospin Tissue extraction kit (NucleoSpin Tissue kit, Macherey-Nagel GmbH & Co. KG., Dueren, Germany). Before the fecal samples were extracted, a purification system (Adiavet Adiafilter (x100), Bio-X Diagnostics S.A., Rochefort, Belgium) was used to achieve the correct sedimentation. After sedimentation, 10 mL of the supernatant was measured on the ADIAFILTER system. For the qPCR run, we used the Adiavet ParaTB Real-Time kit (Bio-X Diagnostics S.A., Rochefort, Belgium), which detects the IS 900 insertion sequence of MAP (47). The running protocol was as follows: 2 min at 50°C, 10 min at 95°C, 30 s at 95°C and 1 min at 60°C during 45 cycles. Ct values below 40 indicated positivity, Ct values between 40 and 45 were rendered “uncertain,” and 45 was the upper limit of detection. Values above the upper detection limit were rendered negative and were substituted with 45 in the statistical analysis.

### 2.3 Statistical analysis

The time period during which the MAP-infected animals could be identified with the highest probability was assessed by calculating the proportion of test-positive animals with 95% CI concerning each method in each sampling interval. McNemar’s test was used to compare the tests in terms of their ability to detect positives among known infected animals. This test considers only the disagreeing results and checks whether one test tends to detect more positives in those cases where the other test returned a negative diagnosis. Results of multiple pairwise comparisons (milk ELISA: serum ELISA; milk ELISA: faecal qPCR; serum ELISA: faecal qPCR) were corrected for false discovery rate. We used Spearman’s correlation testing to evaluate the correlation between serum and milk ELISA S/P outcomes and fecal RT-PCR Ct values.

To investigate whether a distinct pattern of serological or clinical markers would predict the onset of the clinical phase of the disease, separate sets of statistical analyses were conducted for (a) the total number of animals, (b) cows who did not develop clinical symptoms and (c) cows who were culled due to clinical symptoms during the observation period. PCR Ct values, ELISA S/P values (both serum and milk), body condition scores and fecal scores were compared between animals not developing and animals developing clinical symptoms of paratuberculosis. Due to the non-normality of ELISA S/P values and PCR Ct values, the Mann–Whitney test was used to compare groups. Assumptions were tested by exploratory analysis of distributions of outcome parameters. The temporal pattern of changes in S/P and Ct values was explored with nonlinear models, with sampling time taken as a continuous predictor (the last time point of each sampling interval). The level of statistical significance was set at *p* < 0.05 for all tests. All data visualizations and statistical procedures were performed in the R statistical environment (48).

## 3 Results

The number of positive diagnoses obtained with the different diagnostic methods for the total number of animals, animals without and with signs of clinical paratuberculosis, are shown in Tables 1–3, respectively.

**Table 1:** Number of MAP-positive animals out of the total number of animals according to diagnostic method and sampling interval.

Sampling period	Serum ELISA	Milk ELISA	Fecal qPCR
DIM 0–5	33/52 (63.5, 48.9; 76.4)^a^	13/52 (25, 14.0; 38.9)^b^	39/52 (75, 61.1; 86.0)^a^
DIM 10–14	33/52 (63.5, 48.9; 76.4)^a^	27/52 (51.9, 37.7; 66.0)^a^	42/52 (80.8, 67.5; 90.4)^b^
DIM 40–60	40/52 (76.9, 63.2; 87.5)	32/52 (61.5, 47.0; 74.7)	42/52 (80.8, 67.5; 90.4)
DIM 90–120	22/35 (62.9, 44.9; 78.5)	17/36 (47.2, 30.4; 64.5)	17/36 (47.2, 30.4; 64.5)
DIM 180–200	8/33 (24.2, 11.1; 42.3)	3/33 (9.1, 1.9; 24.3)	7/33 (21.2, 8.9; 38.9)
DIM 280–300	13/33 (39.4, 22.9; 57.9)^a^	5/33 (15.2, 5.1; 31.9)^b^	12/33 (36.4, 20.4; 54.9)^a^

The analysis includes the data of all animals. The serum ELISA test result was not obtained for one animal for the period of 90–120 DIM. Results are displayed as number of positives per total and percentages with 95% confidence interval.

^a,b^Different superscripts within a row indicate significant differences between tests in diagnosing known infected animals positive based on pairwise McNemar’s tests (*p* < 0.05). MAP: *Mycobacterium avium* subsp. *paratuberculosis*; DIM: days in milk.

**Table 2:** Number of MAP-positive animals out of the animals not showing signs of clinical paratuberculosis in the observation period (non-culled) according to the diagnostic method and sampling interval.

Sampling period	Serum ELISA	Milk ELISA	Fecal qPCR
DIM 0–5	18/36 (50, 32.9; 67.1)^a^	2/36 (5.6, 0.7; 18.7)^b^	23/36 (63.9, 46.2; 79.2)^a^
DIM 10–14	17/36 (47.2, 30.4; 64.5)^ab^	11/36 (30.6, 16.3; 48.1)^a^	26/36 (72.2, 54.8; 85.8)^b^
DIM 40–60	27/36 (75, 57.8; 87.9)^ab^	17/36 (47.2, 30.4; 64.5)^c^	26/36 (72.2, 54.8; 85.8)^ac^

Results are displayed as number of positives per total and percentages with 95% confidence interval.

^a,b,c^Different superscripts within a row indicate significant differences between tests in diagnosing known infected animals positive based on pairwise McNemar’s tests (*p* < 0.05). Lack of superscripts indicates no significant difference. MAP, *Mycobacterium avium* subsp. *paratuberculosis*; DIM, days in milk.

**Table 3:** Number of MAP-positive animals out of the animals culled after DIM 60 due to clinical manifestation of paratuberculosis (*n* = 16) according to the diagnostic method and sampling interval.

Sampling period	Serum ELISA	Milk ELISA	Fecal qPCR
DIM 0–5	15/16 (93.8, 69.8; 99.8)	11/16 (68.8, 41.3, 88.9)	16/16 (100, 79.4; 100)
DIM 10–14	16/16 (100, 79.4; 100)	16/16 (100, 79.4; 100)	16/16 (100, 79.4; 100)
DIM 40–60	13/16 (81.3, 54.3; 95.9)	15/16 (93.8, 69.8; 99.8)	16/16 (100 79.4; 100)

Results are displayed as number of positives per total and percentages with 95% confidence interval.

Different superscripts within a row indicate significant differences between tests in diagnosing known infected animals positive based on pairwise McNemar’s tests (*p* < 0.05). Lack of superscripts indicates no significant difference. MAP, *Mycobacterium avium* subsp. *paratuberculosis*; DIM, days in milk.

The rate of positive diagnoses varied during lactation, with a higher rate of positivity observed at the beginning and a lower rate of positivity at the end of lactation. The highest rate of positive diagnoses for all methods was observed in DIM 40–60 for the total number of animals (Table 1). The agreement of tests differed between diagnostic techniques in the sampling intervals of DIM 0–5, 10–14, and 280–300. The proportion of positive diagnoses was significantly higher with serum ELISA and fecal RT-PCR in DIM 0–5 than with milk ELISA. However, in DIM 10–14, the proportion of positive diagnoses with fecal RT-PCR was higher than that of both serum and milk ELISA assay. At the time of sampling in 280–300, DIM, fecal RT-PCR, and serum ELISA identified a higher proportion of positive animals than milk ELISA (Table 1).

Exploring these proportions in animals not developing and developing clinical symptoms during the study, it was observed that most animals that later developed clinical symptoms had already tested positive right from the beginning of lactation (Table 3). There were no significant disagreements between methods in any of the sampling periods. Animals that later did not develop clinical symptoms were less likely to be positive at 0–5 DIM and 10–14 DIM (Table 2). The highest positivity rates were observed between 40–60 DIM, which led to the highest positivity rates for the total number of animals observed during this period.

**Figure 1:**
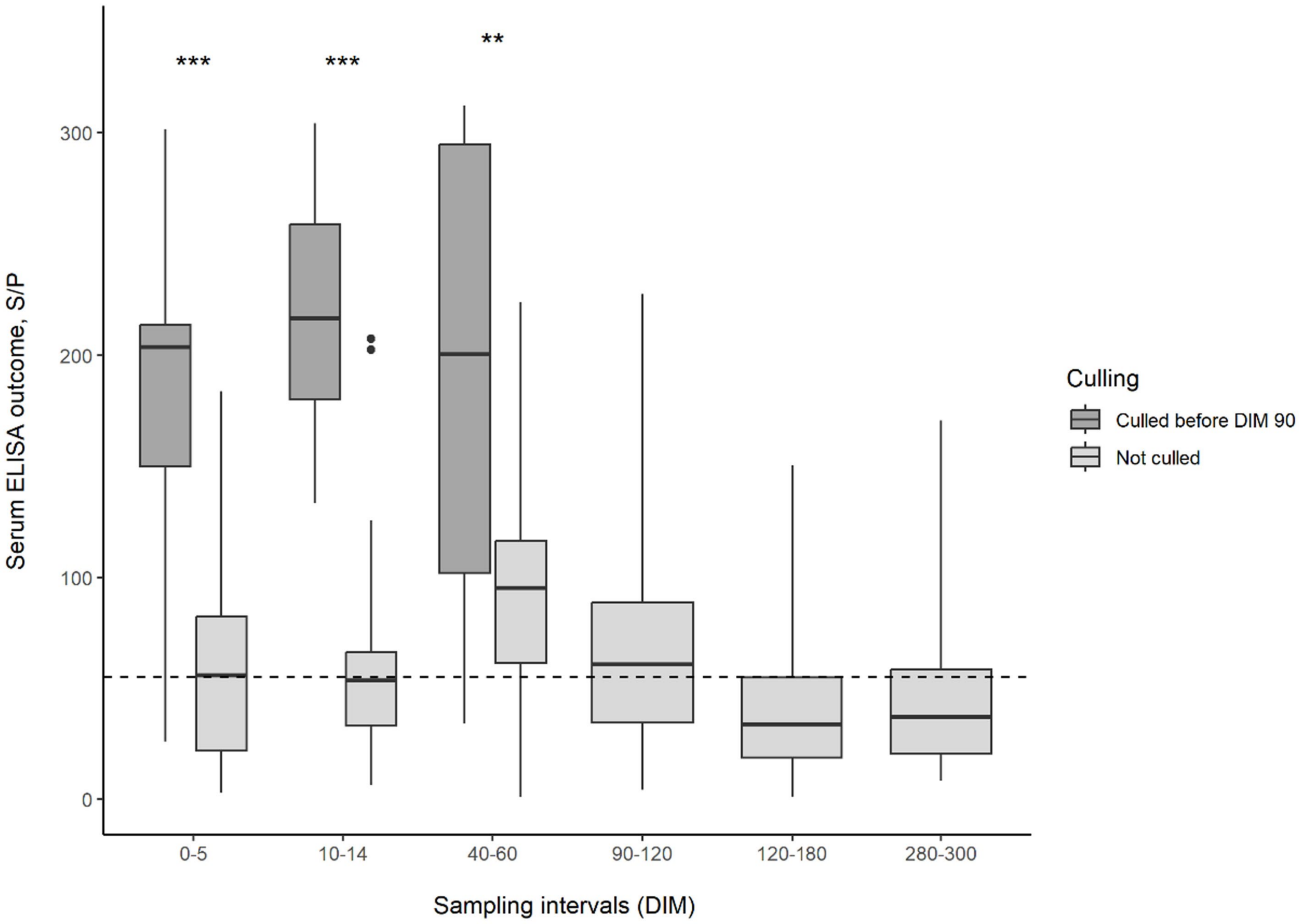
Boxplot of MAP-serum ELISA outcomes for animals culled and not culled between 60–90 DIM and sampling interval. Asterisks indicate significant differences between group medians (*p* < 0.001). The dashed line indicates the threshold for ELISA positivity in serum. MAP, *Mycobacterium avium* subsp. *paratuberculosis*; DIM, days in milk.

**Figure 2:**
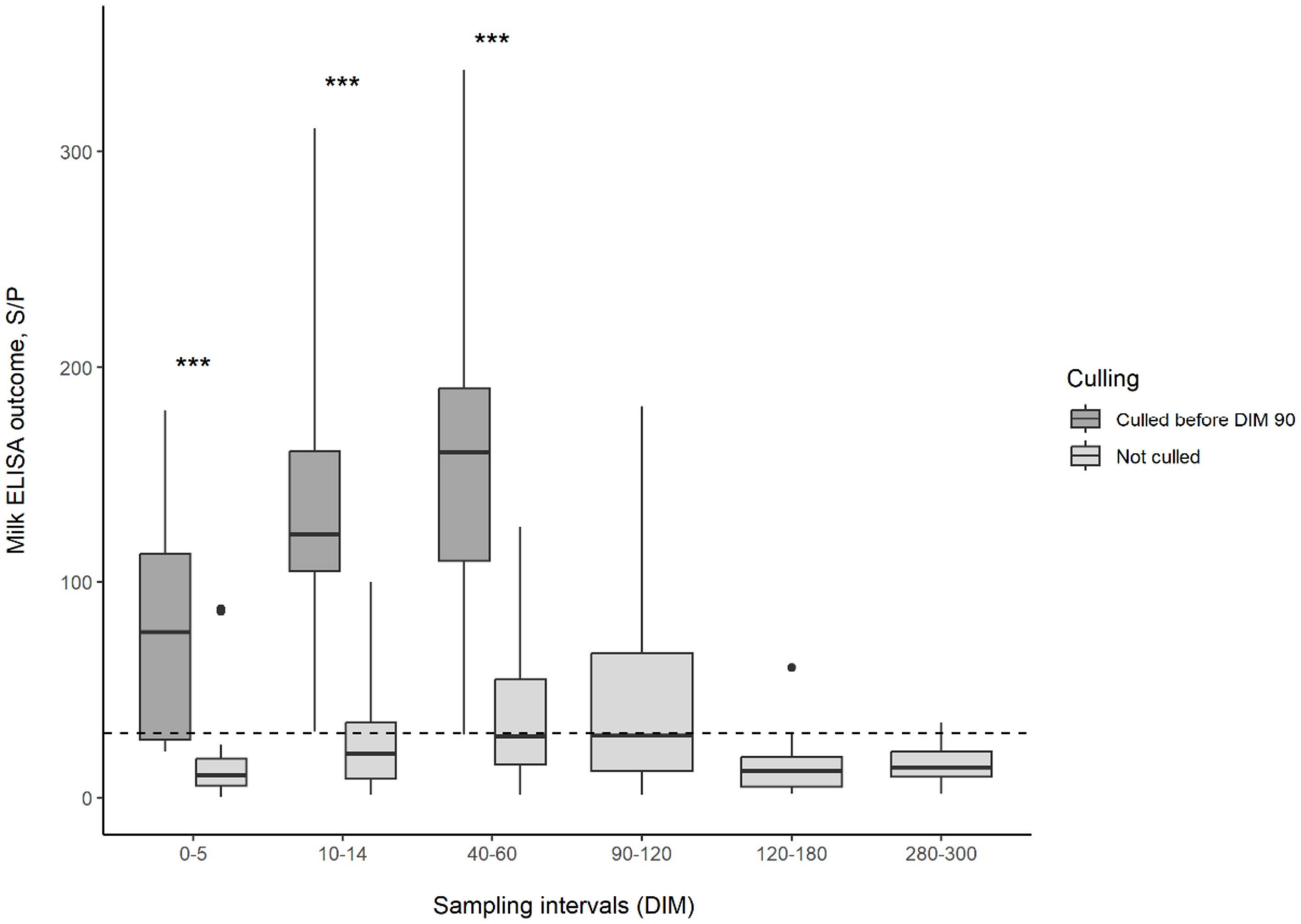
Boxplot of MAP-milk ELISA outcomes for animals culled and not culled between 60–90 DIM and sampling interval. Asterisks indicate significant differences between group medians (*p* < 0.001). The dashed line indicates the threshold for ELISA positivity in milk. MAP, *Mycobacterium avium* subsp. *paratuberculosis*; DIM, days in milk.

**Figure 3:**
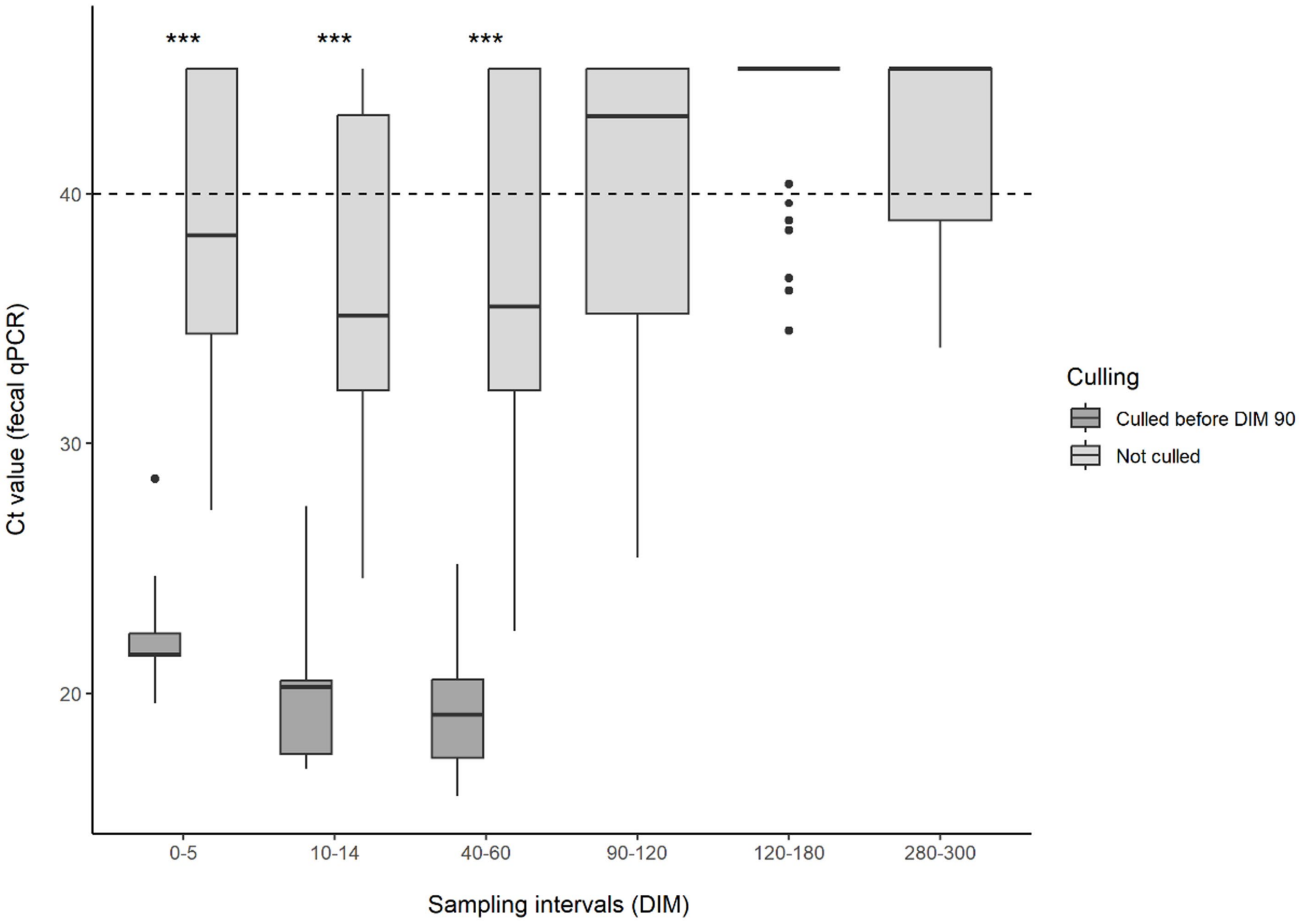
Boxplot of MAP-fecal Ct values for animals culled and not culled between 60–90 DIM and sampling interval. Asterisks indicate significant differences between group medians (*p* < 0.001). The dashed line indicates the threshold for qPCR Ct positivity in feces. MAP, *Mycobacterium avium* subsp. *paratuberculosis*; Ct, cycle threshold; DIM, days in milk.

This distinct difference in the test results of animals developing and not developing clinical symptoms was also observed when comparing test values. The changes in serum or milk ELISA S/P values and feces Ct values during lactation can be observed in Figures 1–3, respectively. Serum ELISA S/P values were significantly higher in all sampling periods in the cows culled between DIM 60–90 than in the non-culled ones (Figure 1). In the non-culled animals, serum ELISA values increase until DIM 40–60, then decrease until lactation ends (Figure 1). A similar pattern is visible in the case of milk ELISA S/P values (Figure 2). The trend is the opposite regarding fecal PCR Ct values (Figure 3). The Ct values of animals culled were significantly lower than those of the non-culled ones. The Ct values of the non-culled animals decrease at the beginning of lactation until DIM 40–60 and then increase.

**Figure 4:**
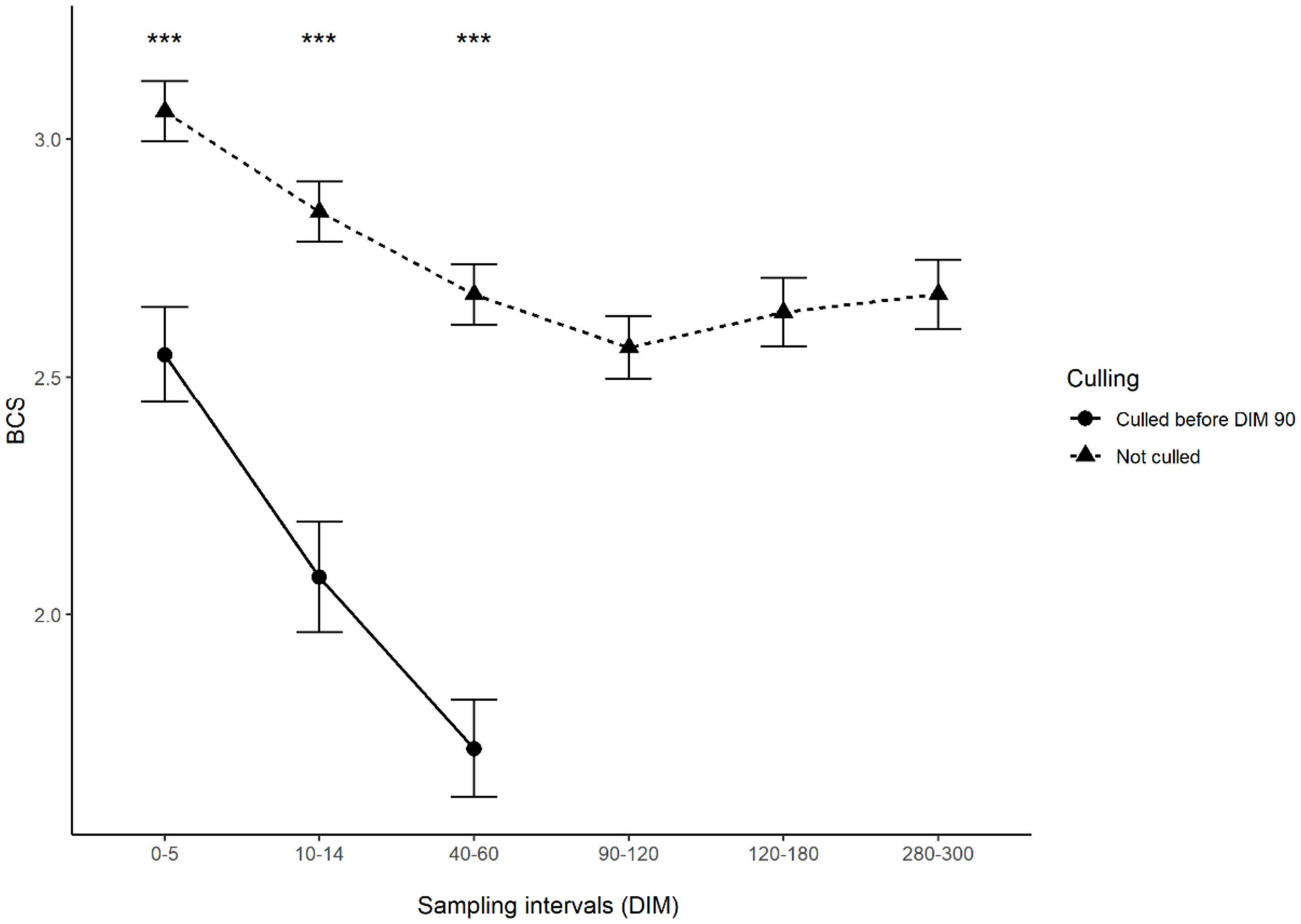
Mean body condition scores of animals culled and not culled between 60 and 90 DIM according to the sampling interval. Error bars represent standard error. Asterisks indicate significant differences between groups (*p* < 0.001). For better interpretation, means are displayed, however, tests were performed on medians.

**Figure 5:**
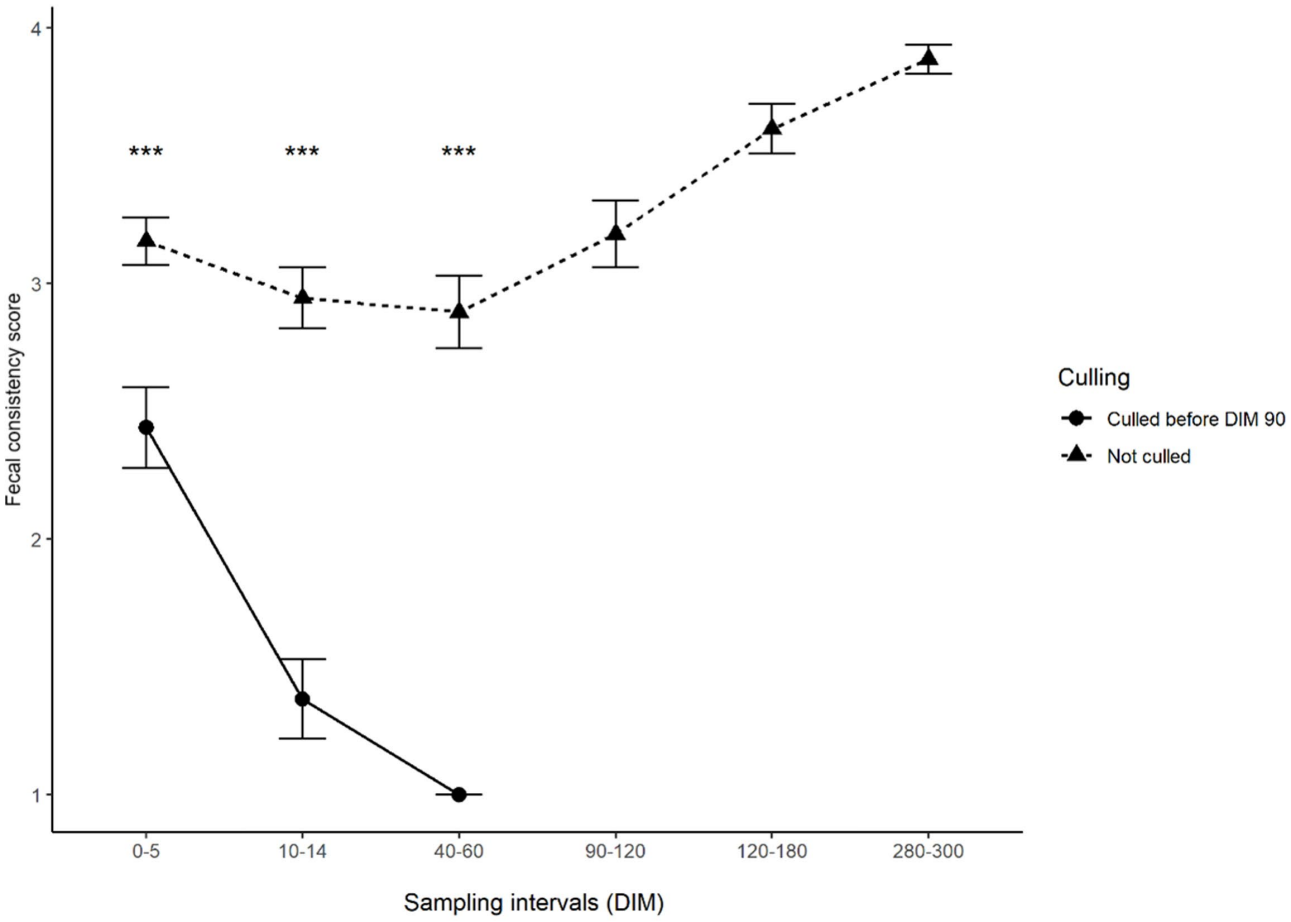
Mean fecal scores of animals culled and not culled between 60 and 90 DIM according to the sampling interval. Error bars represent standard error. Asterisks indicate significant differences between groups (*p* < 0.001). For better interpretation, means are displayed, however, tests were performed on medians.

Figures 4 and 5 show the change in body condition and fecal scores after calving in animals that remained in the study and those that were culled between days 60 and 90. It could be observed that while animals not developing clinical symptoms in the study period had body condition and fecal score in the range that is normal at the beginning of lactation, cows later developing clinical symptoms showed a sharp and continuous decrease in both body condition and fecal consistency, which is indicative of severe diarrhoea.

When investigating the strength of association between ELISA S/P and fecal PCR Ct values (Supplementary Figures 1–7), a similar pattern was observed for the total number of animals, animals not developing clinical signs of paratuberculosis and animals developing clinical signs of tuberculosis. Serum and milk ELISA S/P values showed a significant positive correlation in all sampling intervals. Fecal PCR Ct values and ELISA S/P values were negatively correlated, though the association was insignificant in all sampling intervals. Exploring the nonlinear pattern of the change in S/P and fecal PCR Ct values, the group not developing clinical symptoms and the group manifesting clinical symptoms showed distinctively different patterns of change, with animals culled later showing considerably lower Ct values and higher S/P values (Supplementary Figure 8).

## 4 Discussion

JD is an infectious disease that causes significant economic losses in dairy herds (1). Controlling the disease presents a challenging task to veterinarians caring for the affected herds, as diagnosis is difficult in the early stages of infection. The currently available diagnostic tests have significant limitations; however, establishing testing and management protocols is essential to control the disease (2). Information about the change in the sensitivity of diagnostic tests during lactation is necessary to choose the most suitable time for sampling from a cost-effectiveness point of view. Examining the sensitivity of diagnostic tests in different stages of lactation and the relationship between currently available diagnostic tests can help establish an effective culling program.

Out of the 52 cows selected for our study, 16 were culled due to clinical JD. The JD was diagnosed according to the clinical symptoms, and the farm veterinarians made the culling decision. During the study, the farmers and the vets were not informed about the ELISA and qPCR results. These culled animals can be considered high shedders or progressors (38, 43), while the others, which remained in the study, can be regarded as low shedders. All the culled cows started their lactation with lower body condition and fecal scores, higher ELISA S/P results, and lower qPCR CT values. Based on these results, collecting serum/milk or fecal samples at the beginning of lactation may provide an opportunity to make a rapid culling decision before clinical signs appear.

During the study, the highest positive rate of all three tests fell between days 40–60 of lactation in the total number of animals; however, it was not 100%. This date of DIM is convenient for sampling. In the case of a positive result obtained at the 40–60 DIM sample, the affected cow can be removed from the animals intended for insemination during the voluntary waiting period and leave the herd at the end of the lactation, thus preventing it from becoming a source of infection for the newborn calves at the next calving (17).

Regarding the different tests, the ratio of milk ELISA positivity was high at the beginning of the lactation, as was shown earlier (39), and correlated well with the serum analysis in our case, except right after parturition (DIM 0–5). The low number of positive milk ELISA results at DIM 0–5 can be explained by the high-fat content of colostrum, which decreases the efficiency of the ELISA method (49). Our results partially contradict a study on a large number of animals, according to which the ELISA positivity rate is higher in the case of milk samples taken at the beginning of lactation, while in the case of serum samples, a higher proportion of positivity is shown at the end of lactation (50). However, that study was performed on different animals with no repeated measurements. Our study also demonstrates that antibody levels change during lactation, as shown in previous studies (51). Serum and milk ELISA positivity increased until DIM 40–60, then decreased. Beaver et al. (39) also found that milk ELISA positivity is higher in early lactation. Faruk et al. (42) showed a fluctuation in ELISA patterns during lactation, depending on the level of positivity in the given animal.

The proportion of qPCR-positive samples was the highest at the beginning of lactation until day 60 in the present study. It has been reported that calving, a significant stressor, increases the rate of bacterial shedding (52). On the other hand, Laurin et al. (53) found the sensitivity of qPCR to be higher during the dry period compared to fecal samples collected 14 days after calving. Still, no statistically significant differences were found between the two groups (53). As we did not examine the dry period but only sampled the animals at the end of lactation and drying-off, no data concerning the dry period of the animals enrolled in the study are available. This is a limitation of the study, but we found sampling during the dry period less practical under farm conditions, as the present study aimed to make the sampling schedule as realistic as possible. The probability of qPCR positivity was the lowest at the end of lactation in the study by Laurin (53), similar to our results. Figure 2 shows that the Ct values of the fecal samples at the beginning of lactation were the lowest in the group that did not have to be culled. The Ct value increases as lactation progresses after 60 DIM, indicating that the bacterial shedding was most significant at the beginning of the lactation and then decreased.

Similar to earlier studies, our test results never yielded 100% positivity detection (51). Therefore, repeated sampling at the beginning of lactation is necessary to increase sensitivity as the basis for a culling decision. Furthermore, the results of our study indicate that there is a greater chance of getting a false negative result during the repeated examination towards the end of lactation.

Previous studies showed that the ELISA’s S/P values correlate with bacterial shedding (26). Our study proved that this correlation exists for qPCR Ct values too, during lactation at the sampling times we determined. Overall, as the animals progress toward the clinical phase, the tests show a stronger correlation, according to our results.

When the implementation of testing protocols was discussed in our study, ease of testing was at the forefront of the discussions. We chose the sampling times in such a way as to adapt to the farm practice. According to the protocol, some tests and examinations (e.g., involution checking, pregnancy tests, etc.) are carried out on the farms at each selected time when the animals are handled anyway, so sampling is easy and practical. Added labor to an already busy schedule was seen as something farmers would not easily welcome (54). According to our results, the best time for sampling using serum ELISA was between DIM 40–60, the second-best time for a repeated sample was between DIM 0–14 if high shedders were in the herd, or DIM 90–120 if there were low shedders. If milk ELISA is used, the best time for screening is between DIM 40–60, but DIM 10–14 or DIM 90–120 can also be a suitable second sampling time. Similarly to others (42, 55), serum ELISA gave more accurate results than milk ELISA in our study, although milk is easier to collect. Regarding fecal qPCR, the best sampling time is between DIM 10–14 or DIM 40–60, according to the results of the present study.

## 5 Conclusion

The primary goal of our study was to examine at which stage of lactation and which sample should be taken to detect JD in cows to identify as many positive animals as possible. We found the most suitable period for this to be 40–60 days after calving, which is before insemination, and it can easily be included in a MAP eradication program. Animals later developing and not developing clinical symptoms show distinctively different patterns in test positivity and level of biomarkers. Among the methods, qPCR proved the most reliable testing method during the study, followed by serum ELISA. In all sampling time points in the study, the serum ELISA positivity rate exceeded the positivity rate of milk ELISA. Milk ELISA showed the lowest sensitivity throughout the study. The ELISA S/P values correlated with the fecal qPCR Ct values.

## Data Availability

The raw data supporting the conclusions of this article will be made available by the authors upon request, without undue reservation.
