# Supplementary material for: When is the best time to test paratuberculosis positivity? Observations from a follow-up study in Hungarian dairy herds
Source: Front Vet Sci. 2025 Jun 19;12:1570915. doi: 10.3389/fvets.2025.1570915 (PMC12225305; doi:10.3389/fvets.2025.1570915)

Figure 1. Correlation between serum and milk ELISA outcomes in each sampling interval in in the analysis of total number of animals

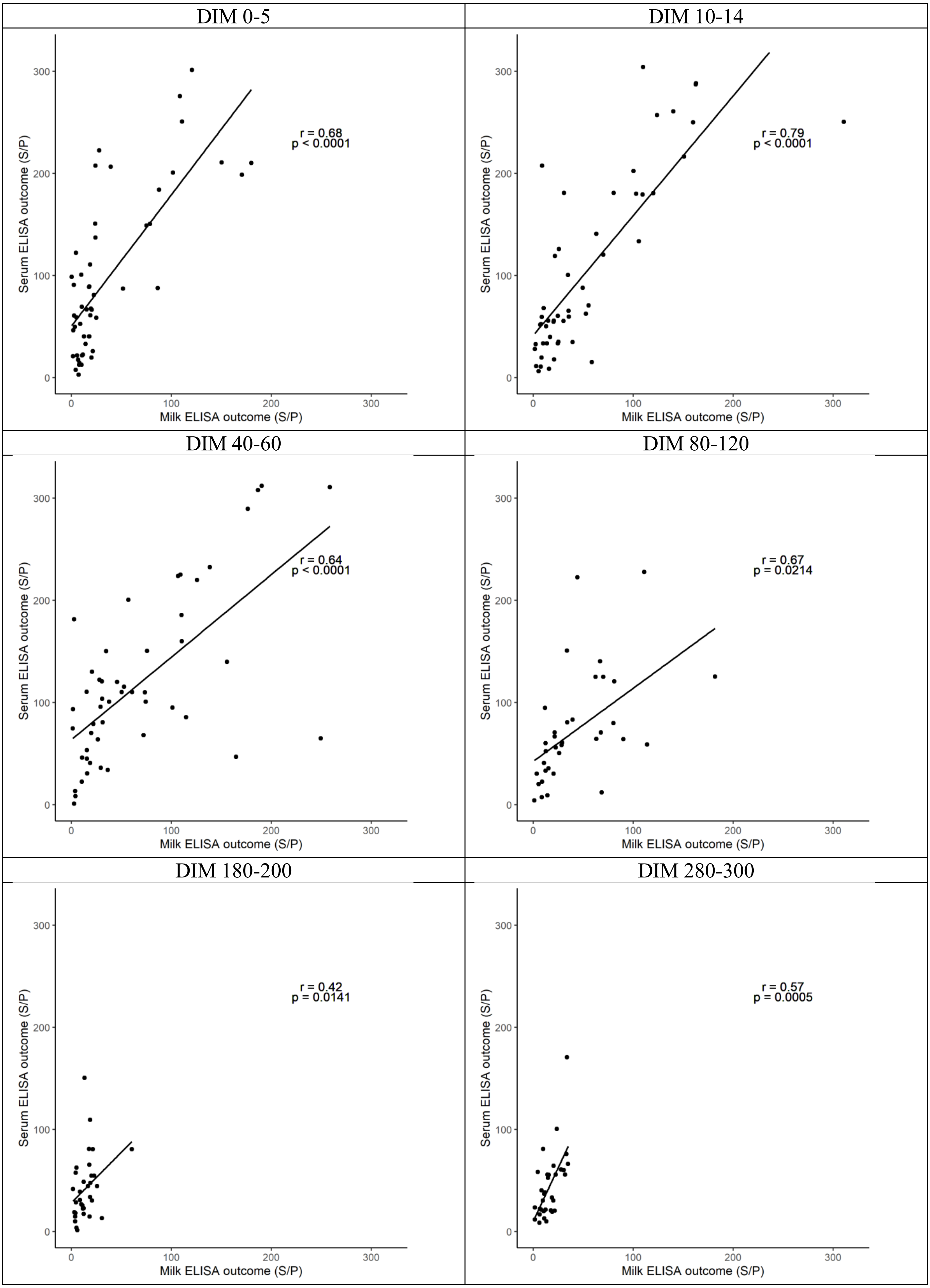

Figure 2. Correlation between serum and milk ELISA outcomes in each sampling interval in the analysis on animals not developing clinical signs of paratuberculosis

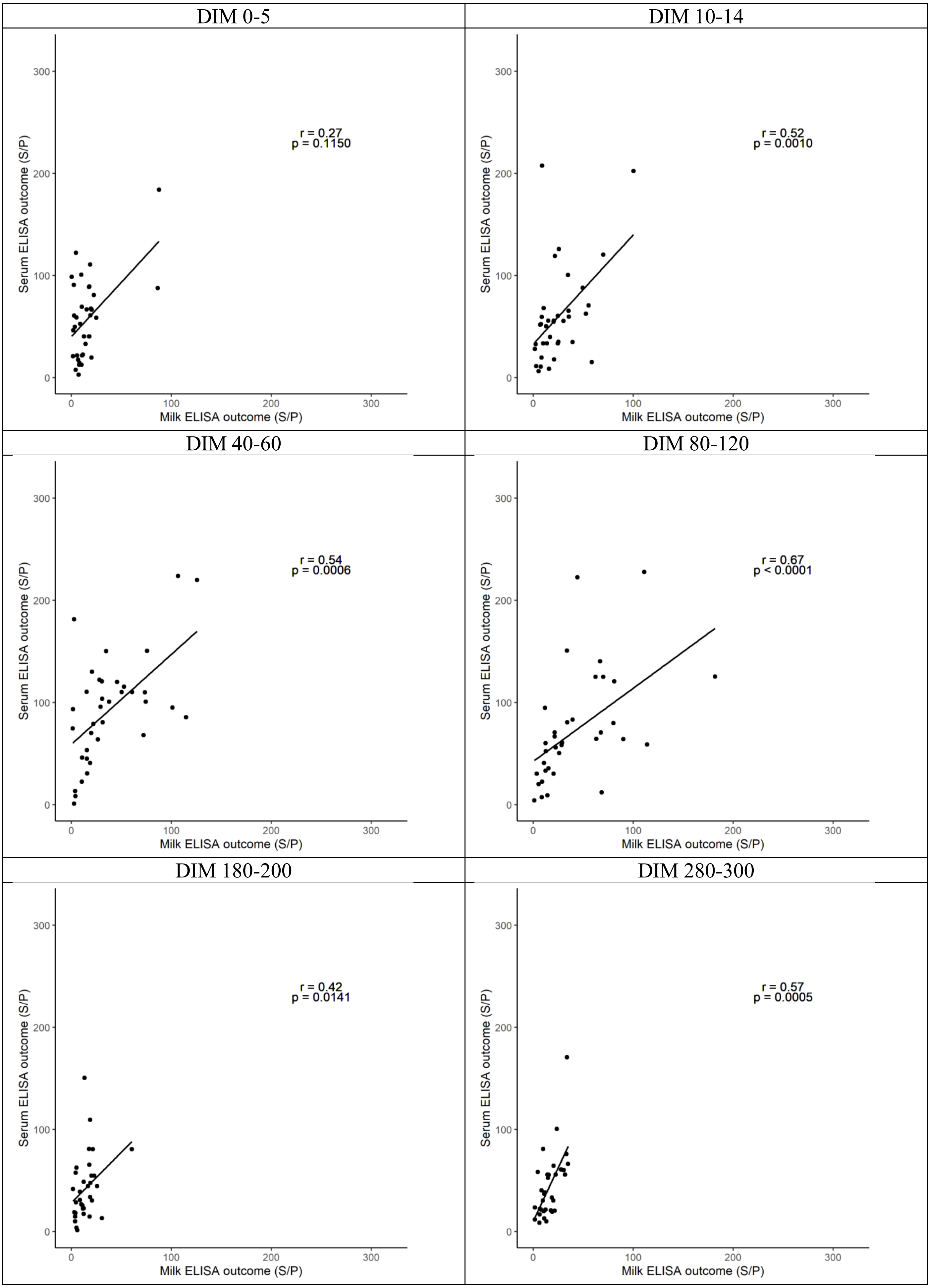

Figure 3. Correlation between fecal Ct values and Serum ELISA outcomes in each sampling interval in the analysis on the total number of animals

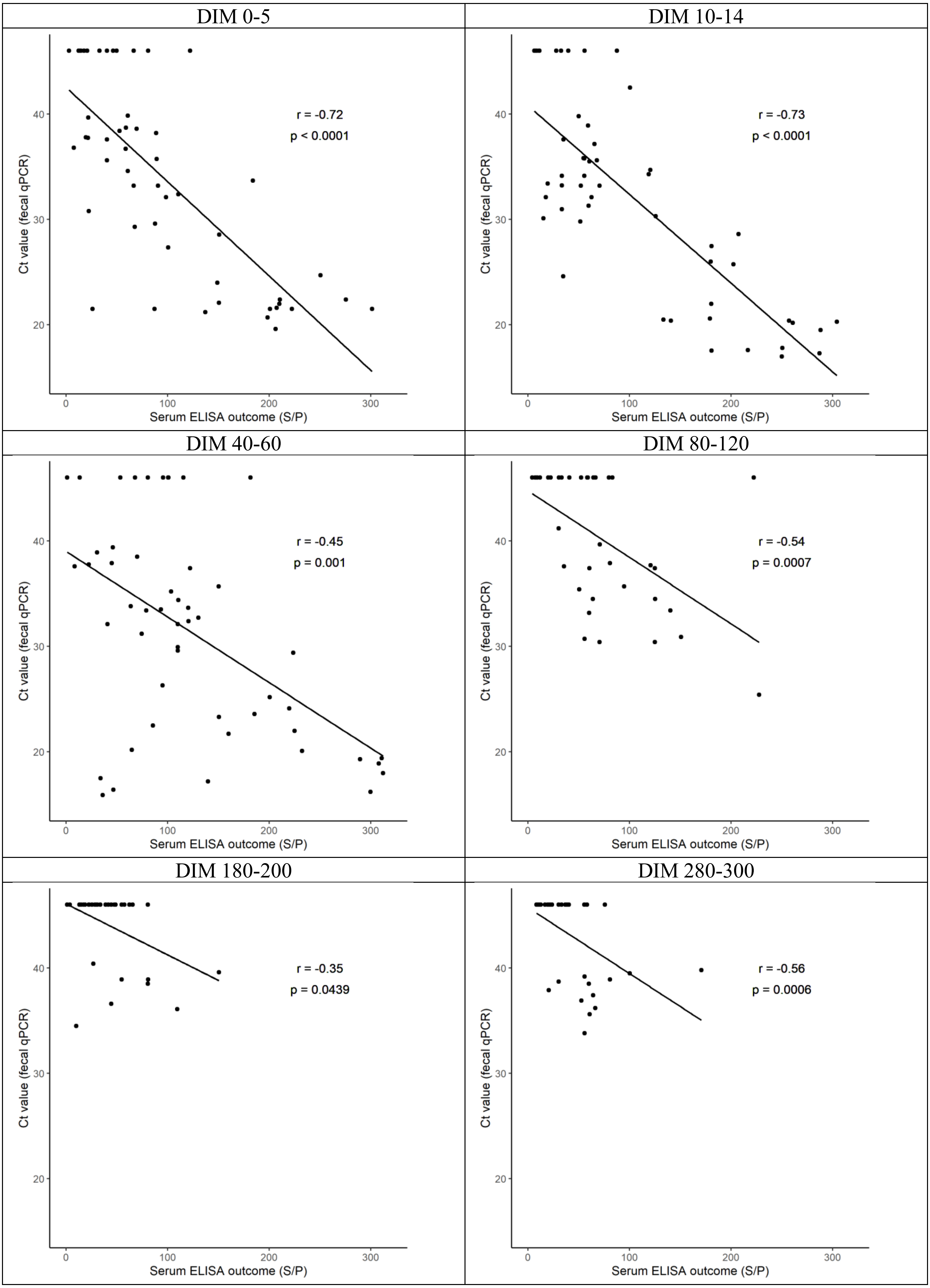

Figure 4. Correlation between fecal Ct values and Serum ELISA outcomes in each sampling interval in the analysis on animals not developing clinical signs of paratuberculosis

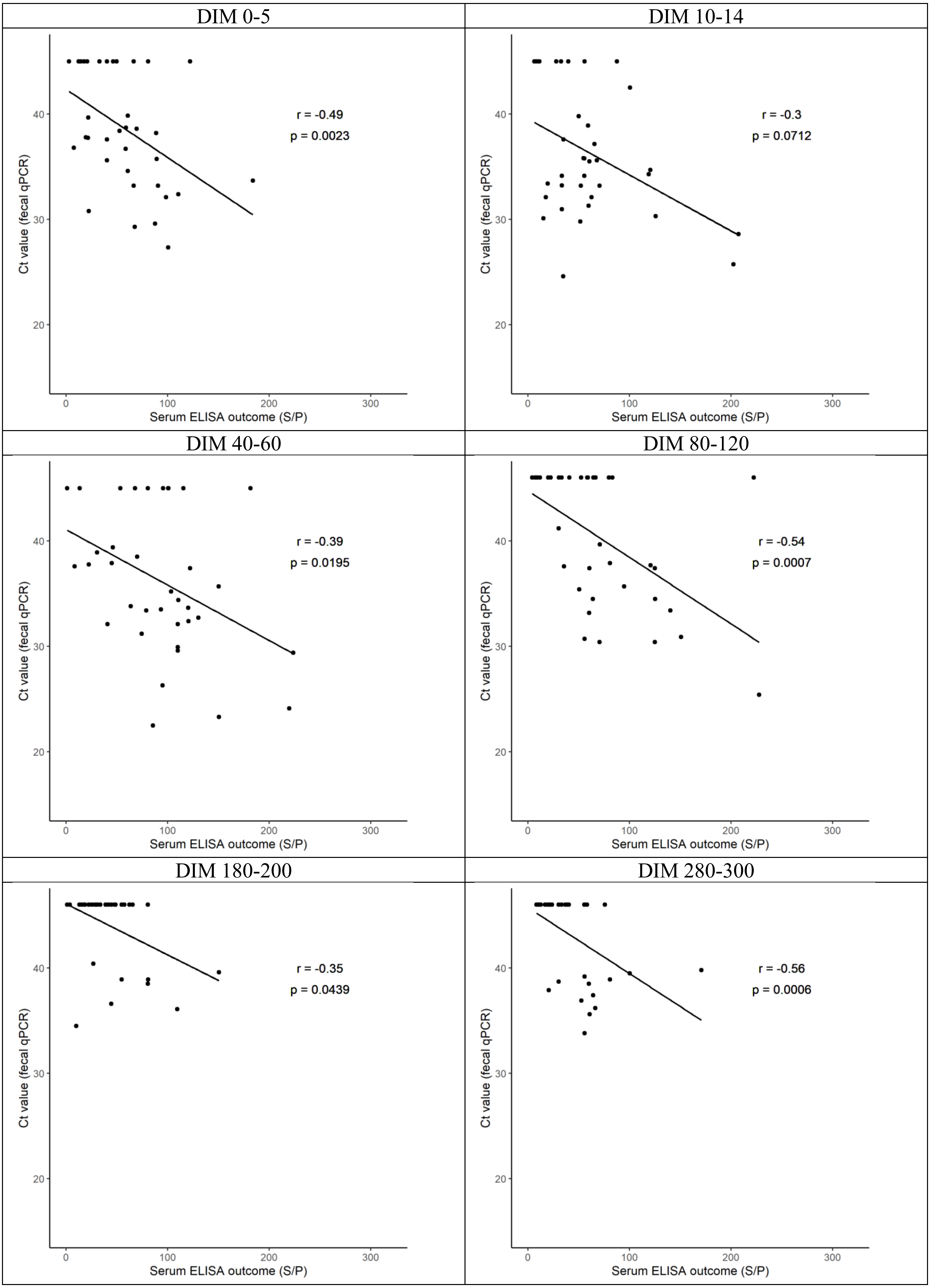

Figure 5. Correlation between fecal Ct values and milk ELISA outcome in each sampling interval in the analysis on the total number of animals

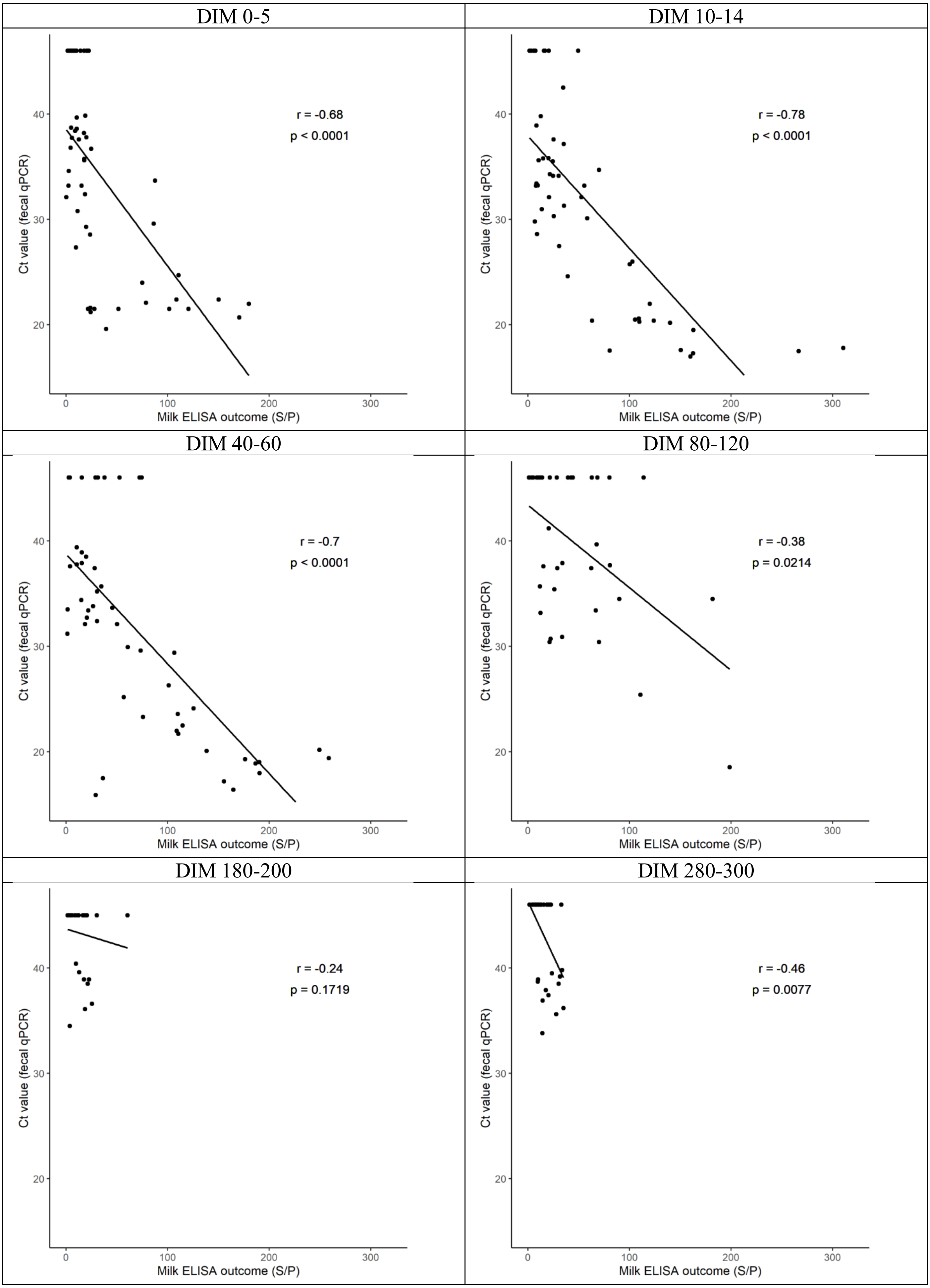

Figure 6. Correlation between fecal Ct values and milk ELISA outcome in each sampling interval in the analysis on the animals not developing clinical signs of tuberculosis.

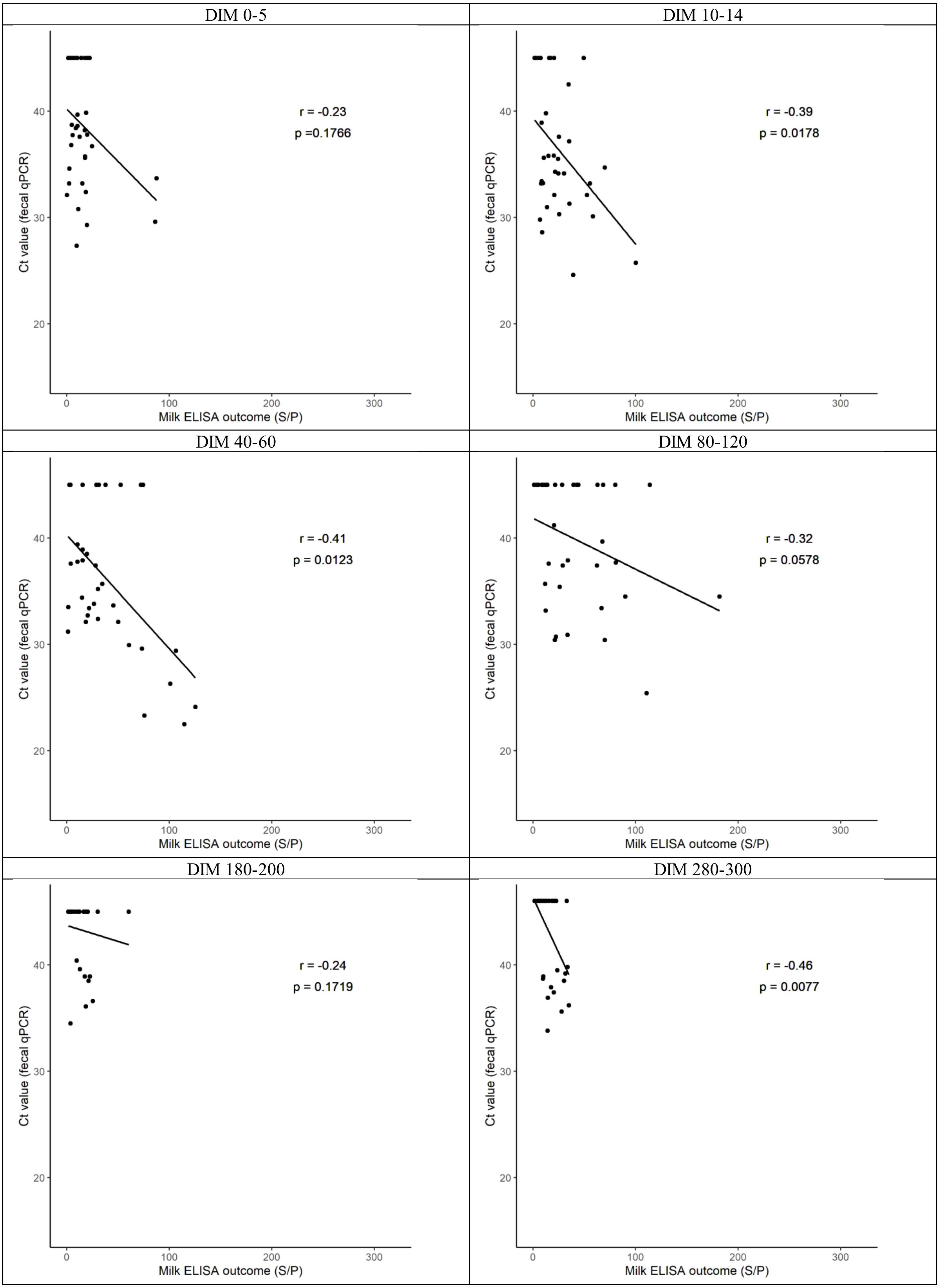

Figure 7. Correlation between biomarkers in each sampling interval in the analysis of the animals developing clinical signs of paratuberculosis.

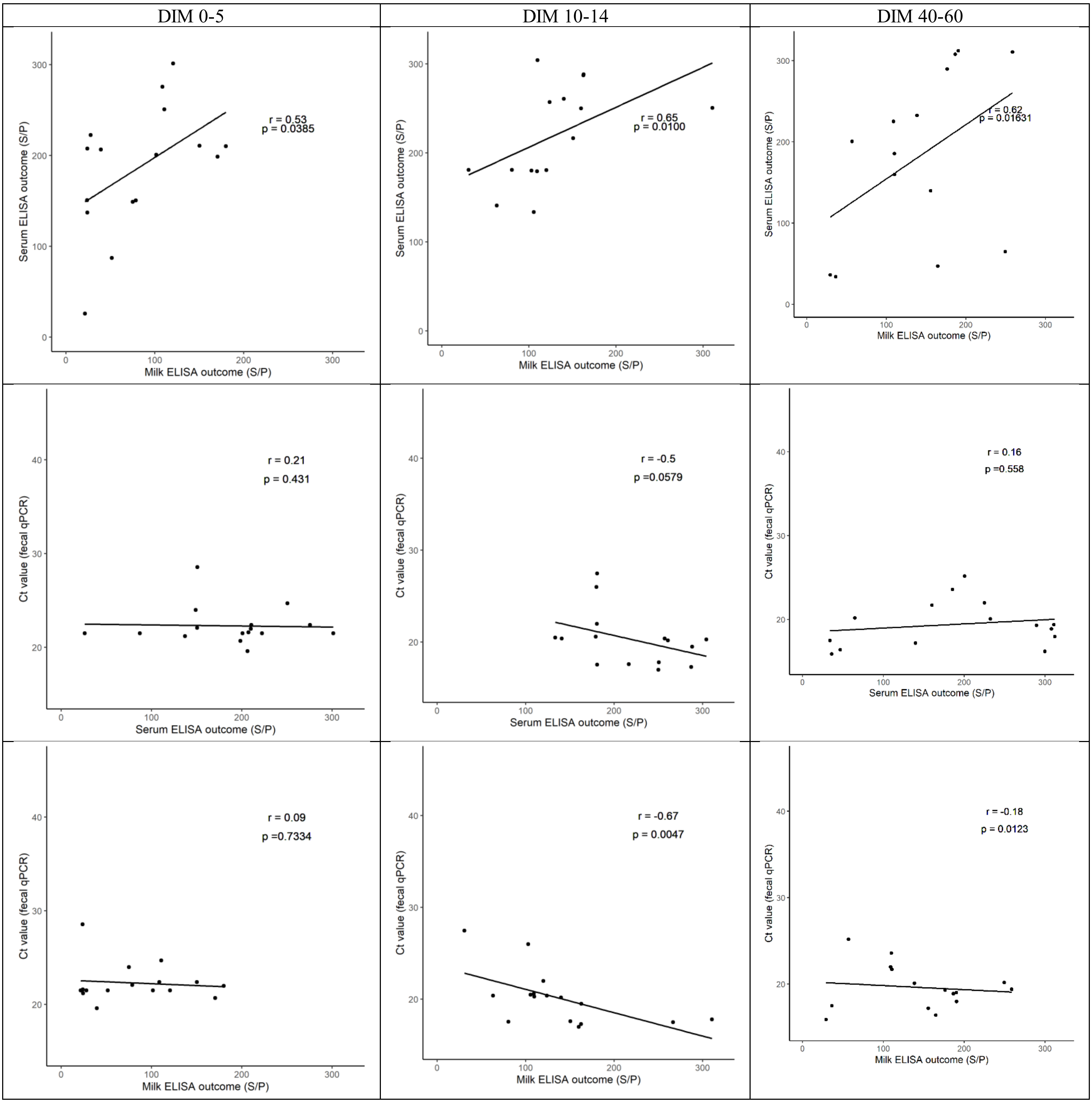

Figure 8. Nonlinear predictions of biomarkers in animals not developing and developing clinical symptoms of paratuberculosis in the observation period

Animals not developing clinical symptoms	Animals developing clinical symptoms

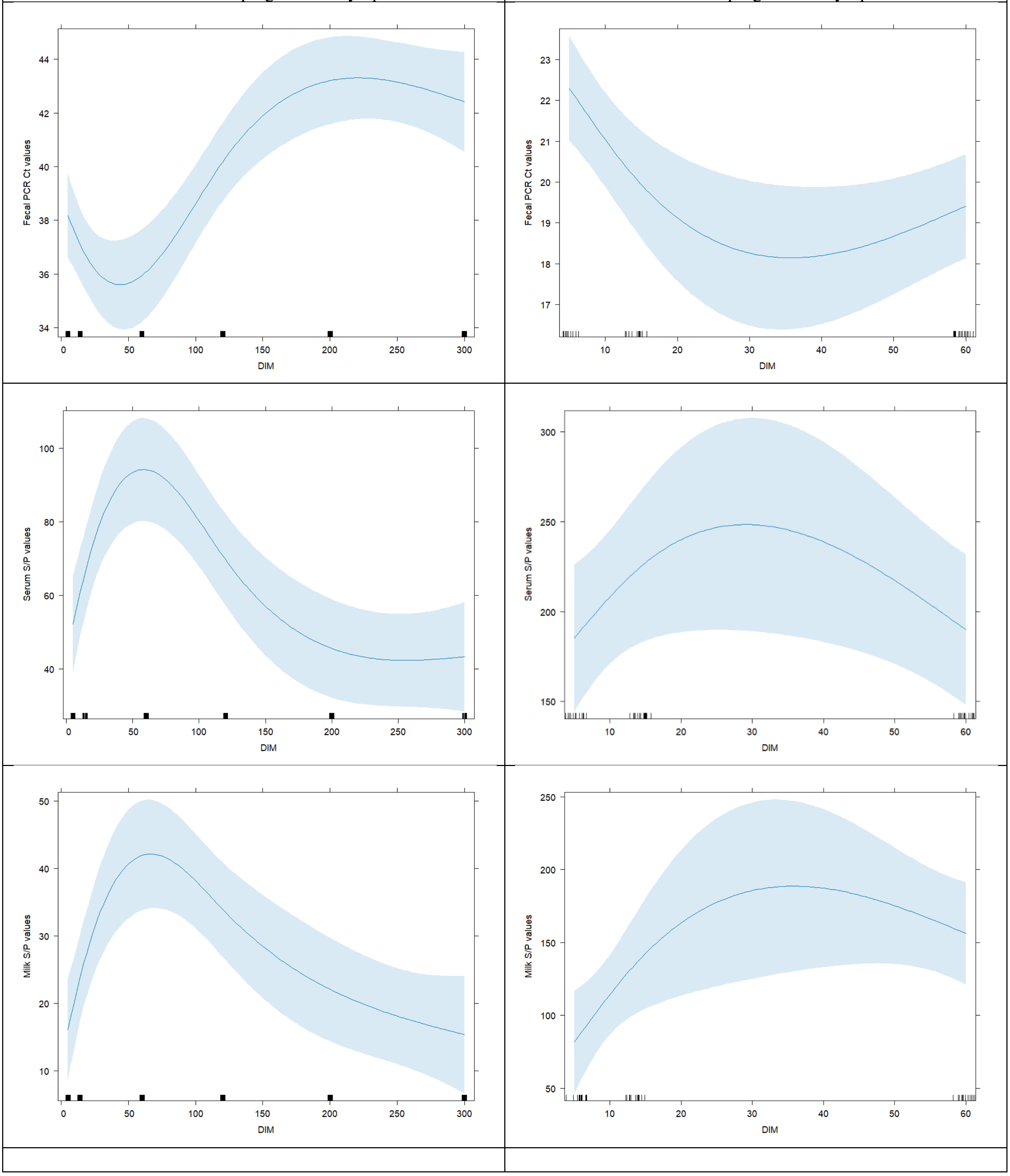

Supplement: Supplementary file 1 [file Data_Sheet_1.pdf]
